# *Actinomyces neuii* in chronic hidradenitis suppurativa: a case series highlighting the value of extended culture

**DOI:** 10.1093/skinhd/vzag031

**Published:** 2026-05-12

**Authors:** Bianca Balji, Louise Lönndahl, Lennart Emtestam, Catarina Sandberg, Hassan Killasli

**Affiliations:** Department of Dermatology, Karolinska University Hospital, Stockholm, Sweden; Department of Dermatology, Karolinska University Hospital, Stockholm, Sweden; Division of Dermatology, Department of Medicine, Solna, Karolinska Institutet, Stockholm, Sweden; Section of Infectious Disease and Dermatology, Department of Medicine, Huddinge, Karolinska Institutet, Stockholm, Sweden; Älvsjö Hudmottagning, Stockholm, Sweden; Section of Infectious Disease and Dermatology, Department of Medicine, Huddinge, Karolinska Institutet, Stockholm, Sweden; Älvsjö Hudmottagning, Stockholm, Sweden

## Abstract

Hidradenitis suppurativa (HS) is a chronic, relapsing inflammatory skin disease characterized by painful nodules, sinus tract formation and scarring. Lesional skin is frequently colonized by diverse aerobic and anaerobic bacteria, with anaerobes such as *Prevotella* spp., *Campylobacter ureolyticus*, *Staphylococcus epidermidis* and *Actinomyces* spp. commonly isolated. *Actinomyces neuii*, a Gram-positive, slow-growing anaerobe, has been previously described only occasionally in deep HS abscesses. We present three cases of treatment-resistant HS in which extended incubation of cultures yielded *A. neuii*. All patients were subsequently treated with long-term amoxicillin (3–6 months), resulting in marked clinical improvement. These findings suggest a potential pathogenic role for *A. neuii* in severe HS. Further research is required to establish its clinical relevance, evaluate optimal antibiotic regimens and monitor for resistance development.

## Teaser

We present three cases of severe hidradenitis suppurativa where extended bacterial culture revealed *Actinomyces neuii* as a potential complicating factor. All patients showed marked clinical improvement following long-term amoxicillin therapy. These findings suggest that infection with *A. neuii* might constitute a complicating factor in patients with treatment resistance in chronic HS.

What is already known about this topic?

- Hidradenitis suppurativa (HS) is a chronic inflammatory disease in which secondary anaerobic bacterial colonization may contribute to more persistent, treatment-resistant disease.
- Slow-growing organisms such as *Actinomyces* may be overlooked with standard culture techniques.

What does this study add?

- Extended culture revealed *Actinomyces neuii* in patients with recalcitrant HS with sinus tracts.
- Prolonged, targeted β-lactam therapy was associated with clinical improvement, supporting extended diagnostics in selected cases.

Hidradenitis suppurativa (HS), also referred to as acne inversa, is a chronic inflammatory skin disorder marked by painful nodules, abscesses, sinus tracts and scarring. HS affects approximately 1% of the population.^1^ It significantly impairs people’s quality of life.^2^ Although primarily considered an autoinflammatory disease, secondary bacterial colonization, particularly with anaerobes, remains clinically relevant.^3–5^ Studies indicate a shift in microbial flora with advancing disease severity, while the role of bacterial biofilms in disease persistence and progression is not fully understood.^4–6^

*Actinomyces* species are Gram-positive, slow-growing anaerobes that are often under-recognized due to insufficient culture duration and prior antibiotic exposure.^7^ Among these, *Actinomyces neuii* has been linked to skin infections and may cause actinomycosis, a chronic condition characterized by sinus tracts and mass-like lesions resembling malignancy.^7,8^ Successful identification requires extended culture of biopsy or purulent samples, ideally prompted by clinical suspicion.^7^

Herein, we present three patients with long-standing HS ­that was unresponsive to conventional therapies in whom *A. neuii* was identified via extended culture. All patients had abcesses and sinus tracts with suspected complicating bacterial infection. MALDI-TOF (matrix-assisted laser desorption/­ionization time-of-flight) mass spectrometry or 16S rRNA gene sequencing were used to identify bacterial species.

## Case report

### Patient 1

A 49-year-old woman, who was otherwise healthy except for asthma, with a long-standing history of HS (Hurley stage III) affecting the groin and lower abdomen had a suboptimal response to oral acitretin and topical treatment. Painful nodules with purulent discharge persisted. Routine culture revealed *Staphylococcus lugdunensis*, while extended incubation isolated *A. neuii*. She was then treated with amoxicillin, initially at a lower dose of 1.5 g daily in combination with clavulanic acid. Thereafter, treatment shifted to amoxicillin 2.25 g daily for 3 months, leading to near-complete remission (Table 1).

**Table 1:** Clinical characteristics, microbiology findings and outcomes in three patients with hidradenitis suppurativa and *Actinomyces neuii*

Patient	Age at treatment (years)	Sex	BMI (kg m^–2^)	History of smoking	Heredity	Hurley stage	Disease duration	Location	Previous treatment	Bacterial culture	Antibiotic treatment	Treatment duration	Adjuvant therapy	Response after therapy
1	49	F	27.1	Yes	Yes	III	> 10 years	Crena ani, thighs, back, face and suprapubic area	Systemic: acitretin, clindamycin, flucloxacillin, tetracycline, zinc. Topical: azelaic acid, potassium permanganate	*Staphylococcus lugdunensis*, *A. neuii* (extended culture time)	Amoxicillin 750 mg, 3 times daily. Total daily dose 2250 mg	3 months	Systemic: acitretin 30 mg daily. Topical: clindamycin. Intralesional: triamcinolone acetonide	Near-complete remission
2	53	F	38.8	Yes	No	II	> 10 years	Perineum, inguinal areas, inner thighs	Systemic: flucloxacillin, spironolactone, zinc. Topical: betamethasone + clioquinol. Intralesional: triamcinolone acetonide	*Actinotignum schaalii*, *A. neuii* (extended culture time)	Amoxicillin 750 mg, 3 times daily. Total daily dose 2250 mg	6 months	Systemic: metformin. Topical: betamethasone + clioquinol, potassium permanganate	Near-complete remission
3	41	F	37.3	Yes	–	II	> 10 years	Axillae, inner thighs, inguinal areas	Systemic: clindamycin, tetracycline. Topical: azelaic acid, potassium permanganate. Intralesional: triamcinolone acetonide	*A. neuii* (extended culture time)	Amoxicillin 1500 mg, 3 times daily. Total daily dose 4500 mg	3 months	Topical: potassium, permanganate, azelaic acid. Intralesional: triamcinolonacetinid injections	Complete remission

BMI, body mass index; F, female.

### Patient 2

Patient 2 was a 53-year-old woman with chronic HS (Hurley stage II) in the perineal and thigh regions, which was resistant to topical and systemic therapies; she was otherwise healthy. Extended culture identified *A. neuii* and *Actinotignum schaalii*. She received amoxicillin 2.25 g daily for 3 months, with substantial improvement. Residual lesions were surgically excised with good outcomes.

### Patient 3

A 41-year-old woman with HS (Hurley stage II), who was otherwise healthy, presented with axillary and groin abscesses that were unresponsive to multiple topical and systemic regimens. Extended culture revealed *A. neuii*. Treatment with higher-dose ampicillin (4.5 g daily) due to more advanced disease with deeper abcesses and scarring for 6 months led to complete remission.

## Discussion

HS is primarily an autoinflammatory condition, but superinfection with specific microbes can exacerbate disease or affect disease progression.^4–6^ The involvement of *A. neuii*, although rarely reported, may represent an under-recognized complicating factor in chronic, treatment-resistant HS.^8,9^ All three patients in our series showed clear clinical benefit from prolonged β-lactam therapy following the identification of *A. neuii*.

While our findings in these three patients do not establish a definitive causal relationship between *Actinomyces* and HS pathogenesis, these observations support a potential pathogenic or exacerbating role rather than mere colonization. Firstly, the organism was consistently isolated from chronic, refractory lesions when extended incubation was performed. Secondly, the clinical response to prolonged antibiotic therapy targeting *Actinomyces* suggests microbial involvement in maintaining inflammation in these reported patients. Finally, the chronic sinus-forming phenotype observed in our patients resembles classical actinomycosis, a granulomatous infection known for its indolent course.^7^

Nevertheless, it remains possible that *Actinomyces* could act as an opportunistic colonizer in the altered microenvironment of HS. The distinction between infection and colonization is challenging in such chronic inflammatory disorders, and further studies are warranted. In addition, antibiotics given previously might further have altered the microbiome in these patients.

Isolation of *A. neuii* requires extended culture, and clinicians should indicate suspicion to microbiology services to ensure appropriate processing. Awareness of this organism’s potential role may facilitate more tailored diagnostic routines and therapeutic approaches in recalcitrant HS.

In summary, while causality cannot be proven, our findings highlight that extended culture techniques can reveal overlooked pathogens such as *A. neuii*, and that targeted antibiotic therapy may lead to meaningful clinical improvement. Therefore, we suggest that clinicians consider *Actinomyces* infection as a possible contributor to treatment-resistant HS.

## Data Availability

The data underlying this article will be shared on reasonable request to the corresponding author.

## References

[1] Sabat R, Alavi A, Wolk K et al Hidradenitis suppurativa. Lancet 2025; 405:420–38. 39862870 10.1016/S0140-6736(24)02475-9

[2] Chernyshov PV, Finlay AY, Tomas-Aragones L et al Quality of life in hidradenitis suppurativa: an update. Int J Environ Res Public Health 2021; 18:6131. 34204126 10.3390/ijerph18116131 PMC8201351

[3] Riverain-Gillet É, Guet-Revillet H, Jais JP et al The surface microbiome of clinically unaffected skinfolds in hidradenitis suppurativa: a cross-sectional culture-based and 16S rRNA gene amplicon sequencing study in 60 patients. J Invest Dermatol 2020; 140:1847–55. 32339539 10.1016/j.jid.2020.02.046

[4] Sartorius K, Killasli H, Oprica C et al Bacteriology of hidradenitis suppurativa exacerbations and deep tissue cultures obtained during carbon dioxide laser treatment. Br J Dermatol 2012; 166:879–83. 22098253 10.1111/j.1365-2133.2011.10747.x

[5] Wark KJL, Cains GD. The microbiome in hidradenitis suppurativa: a review. Dermatol Ther 2021; 11:39–52. 10.1007/s13555-020-00465-w PMC7859000 33244661

[6] Lelonek E, Bouazzi D, Jemec GBE, Szepietowski JC. Skin and gut microbiome in hidradenitis suppurativa: a systematic review. Biomedicines 2023; 11:2277. 37626773 10.3390/biomedicines11082277 PMC10452269

[7] Zelyas N, Gee S, Nilsson B et al Infections caused by *Actinomyces neuii*: a case series and review of an unusual bacterium. Can J Infect Dis Med Microbiol 2016; 2016:6017605. 27366175 10.1155/2016/6017605 PMC4904567

[8] Nedomansky J, Weiss D, Willinger B et al Acne inversa complicated by *Actinomyces neuii*. Infection 2016; 44:247–9. 26129687 10.1007/s15010-015-0814-6

[9] Bonifaz A, Fernández-Samar D, Tirado-Sánchez A et al Hidradenitis suppurativa associated with actinomycosis owing to *Actinomyces meyeri*. Br J Dermatol 2021; 184:e123–4. 33140414 10.1111/bjd.19600

